# Treatment with Umbilical Cord Blood Platelet Lysate Gel Improves Healing of Diabetic Foot Ulcer

**DOI:** 10.3390/jcm13051310

**Published:** 2024-02-26

**Authors:** Vaia Lambadiari, Aikaterini Kountouri, Fοteini Psahoulia, Georgia-Angeliki Koliou, Andreas Lazaris, Efstathios Michalopoulos, Panagiotis Mallis, Emmanouil Korakas, Ioanna Eleftheriadou, Konstantinos Balampanis, Markos Sarris, Panagiotis Tsirigotis, George Geroulakos, Catherine Stavropoulos-Giokas, George D. Dimitriadis, Nikolaos Tentolouris

**Affiliations:** 1 Second Department of Internal Medicine, Attikon University Hospital, Medical School, National and Kapodistrian University of Athens, 12462 Athens, Greece; katerinak90@hotmail.com (A.K.); georgelak@hotmail.com (G.-A.K.); mankor-th@hotmail.com (E.K.); kostasbalabanis@gmail.com (K.B.); panagtsirigotis@gmail.com (P.T.); 2 Department of Vascular Surgery, Attikon University Hospital, 12462 Athens, Greece; faiyps@gmail.com (F.P.); andreaslazaris@hotmail.com (A.L.); ggeroulakos@med.uoa.gr (G.G.); 3 Hellenic Cord Blood Bank, Biomedical Research Foundation Academy of Athens, 11527 Athens, Greece; smichal@bioacademy.gr (E.M.); pmallis@bioacademy.gr (P.M.); cstavrop@bioacademy.gr (C.S.-G.); 4 First Department of Propaedeutic Internal Medicine, Diabetes Centre, Laiko General Hospital, Medical School, National and Kapodistrian University of Athens, 11527 Athens, Greece; joeleftheriadou@yahoo.gr (I.E.); ntentolouris@yahoo.gr (N.T.); 5 Health and Social Care Management, University of West Attica, 12241 Athens, Greece; msaris@gmail.com; 6 Sector of Medicine, Medical School, National and Kapodistrian University of Athens, 11527 Athens, Greece; gdimitr@med.uoa.gr

**Keywords:** diabetic foot, diabetic foot treatment, platelet rich plasma, umbilical cord blood, platelet lysate

## Abstract

Background: This study was conducted to examine the hypothesis that umbilical cord blood platelet lysate (UCB-PL) gel has a significant impact on the healing rate of DFU. Μethods: In this open-labeled, randomized controlled trial, 110 patients were randomized to treatment with UCB-PL gel (UCB-PL group, n = 52) every three days for one month or dressing with normal saline (control group, n = 58). All participants were followed up for 20 weeks post treatment. Ulcer surface area was assessed with the imitoMeasure application at two, four, and six weeks, and two, four and six months. This study’s main outcome was the reduction in ulcer size over the six-month study period. Results: The mean ulcer area at baseline was 4.1 cm^2^ in the UCB-PL group and 1.7 cm^2^ in the control group. At six months post treatment, patients on the UCB-PL treatment displayed a significant reduction in ulcer size compared to baseline 0.12 (0–8.16) in contrast to a more modest change in the control group 1.05 (0–24.7). The ulcer area was decreased at the end of the study in 40 patients (97.6%) in the UCB-PL group and 27 (73%) in the control group (Fisher’s *p* = 0.002). Conclusions: The application of UCB-PL gel in DFU resulted in a significant reduction in ulcer size compared to regular saline dressing.

## 1. Introduction

Diabetic foot is one of the most common, costly and severe complications of diabetes with a remarkable personal, economic and social impact. In most developed countries, the annual incidence of diabetic foot ulcer (DFU) is estimated at 2% [1]. Patients with DFU are susceptible to infection and the healing process is often complicated by the coexistence of neuropathy and vascular disease [2,3]. An estimated 20% of lower-extremity infections will result in amputation. Amputation in people with diabetes is 10 to 20 times more common than in people without diabetes and the risk increases with age and diabetes duration [1].

The goal of ulcer treatment is to entirely heal the wound as expeditiously as possible. The standard treatments for DFU include wound cleansing, necrotic tissue debridement, following revascularization if required, mechanical off-loading, infection management and local ulcer care [4,5]. However, certain risk factors such as infection, tissue hypoxia and repeated trauma commonly contribute to poor wound healing with conventional treatment modalities [6,7]. 

Difficult-to-heal DFU persists for months or years, requiring additional advanced wound care therapies for adequate healing [8,9]. Cellular therapy has been a breakthrough in the treatment of DFU as it is based on the presence of various cytokines and growth factors in blood components and platelet [5]. Platelet-rich plasma (PRP) is an emerging cellular treatment which has gained tremendous interest as a potential adjunctive therapy to conventional care in wounds/ulcers with a low healing ability [10,11,12,13]. However, its clinical application is restricted to platelet gels obtained from autologous peripheral blood, with the significant subsequent limitation being the need for repeated blood collections. This procedure might pose practical difficulties for specific patients, such as those with mobility issues. Therefore, there is a need to produce a large amount of PRP sufficient for broad clinical use. Human umbilical cord blood (UCB) is a promising PRP production alternative with great potential for research and therapeutic purposes [14,15,16]. It has recently been shown that PRP derived from UCB has a similar or even improved effectiveness compared to PRP from peripheral blood in promoting cell growth and differentiation, making it an interesting alternative to treating tissue lesions [15,16].

Platelet lysate (PL) is a novel hemoderivative material which contains increased levels of growth factors, such as epidermal growth factor (EGF), vascular endothelial growth factor (VEGF), fibroblast growth factor, insulin-like growth factor-1, interleukins and interferons [17]. Its relatively easy and cost-effective preparation and its temperature resistance make it a viable alternative product. Data from randomized controlled trials evaluating the implementation of cord blood platelet lysate in chronic wounds are scarce. Especially in the field of diabetic foot ulcers, most data derive from the use of autologous PRP. This is one of the first randomized controlled studies to evaluate the efficacy and safety of treatment with PL derived from UCB compared to regular dressing with saline in DFU.

## 2. Materials and Methods

### 2.1. Study Population

**Figure 1:**
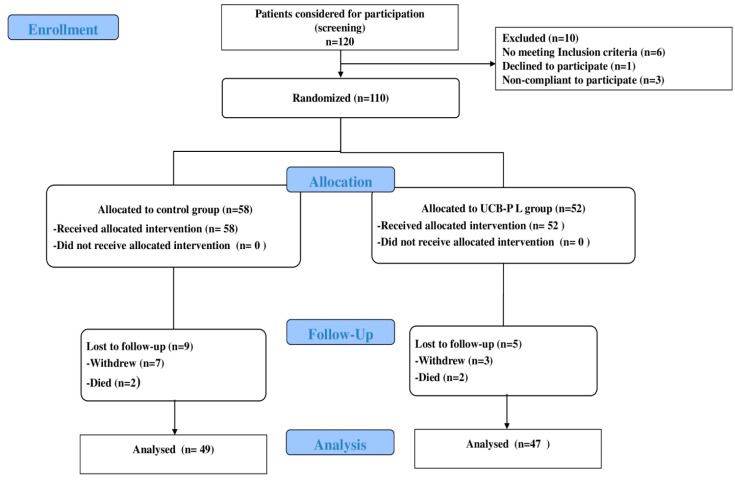
Consort flow diagram showing the progress through the phases of the trial.

We conducted a prospective, open-labeled, randomized, controlled study. Overall, 120 patients were enrolled in this study. Of these, 110 patients met the inclusion/exclusion criteria of this study and were randomized to receive UCB-PL gel (n = 52) or regular dressing with normal saline (n = 58) (Figure 1). The inclusion criteria for this study were subjects with type 1 diabetes or type 2 diabetes, age>18 years old, patients with non-healing DFU, defined as ulcers’ duration > 6 weeks or ulcers with a surface area reduction < 30% over a 7-day period despite optimal standard of care (SOC) management, foot ulcer located on the plantar, medial, or lateral aspect of the foot (including all toe surfaces), an ulcer with area (length x width) measurement < 30 cm^2^, neuropathic, ischemic, neuropathic/ischemic and ulcers after amputation, and absence of infection. The cause of amputation was ischemia-related gangrene, and the operation was combined with revascularization of the limb.

Prior to enrollment, patients with ischemic ulcers had undergone revascularization. Exclusion criteria were pregnancy, venous ulcers, presence of infection, exposure of bone, muscle, ligaments, or tendons and tunneling. In all patients, we recorded age, sex, comorbidities and concomitant medications. The study investigator explained the entire treatment and follow-up procedure to all patients who met the inclusion criteria. All participants were instructed on ulcer care and offloading. All participants signed an informed consent form prior to any procedure included in the study protocol. This study was approved by the institutional ethical board of University General Hospital “Attikon” (411/05-06-2019). All methods were conducted according to relevant guidelines and regulations (Declaration of Helsinki). 

### 2.2. Preparation of UCB-PL Gel 

#### 2.2.1. Collecting Cord Blood and Evaluating Suitability for Non-Transplant

All cord blood units used in this study were collected by specialized midwives after a signed informed consent form was obtained by the mothers before gestation. The cord blood units were processed immediately after the reception at the Hellenic Cord Blood Bank (HCBB) of the Biomedical Research Foundation Academy of Athens (BRFAA), while the time between the collection and the processing did not exceed 48 h. A 0.5 mL blood sample was collected from each cord blood unit and was evaluated in a hematological analyzer (Sysmex XS1000i, Sysmex Europe, Norderstedt, Germany). If the cord blood unit did not fulfill the criteria for processing, cryopreservation and release outlined by FaCT-NetCORD [18], then it was used for UCB-PL production.

#### 2.2.2. UCB-PL Preparation 

Cord blood units were centrifuged at 865× *g* for 15 min at room temperature. The isolation of the PRP fraction was performed via the plasma extractor Mikromatik (LMB TechnologieGmBH, Germany). Then, a second centrifugation followed at 2500× *g* for 15 min to obtain the platelet concentrate. Τhe concentrated PRP products were placed at −80 °C in cryostorage bags (ADVANTIX-SH 50B, Milan, Italy), for at least 48 h. Then, the concentrated PRPs were rapidly thawed in a water bath at 37 °C to form the cord platelet lysate products. Methods for collecting the cord blood and the UCB-PL preparation have been previously described in detail in the literature [19].

#### 2.2.3. UCB-PL Gel Activation before Application

In order to prepare the investigational product, 20% calcium gluconate (Manufacturer) was added to UCB-PL to form a platelet gel ready for application on the wound. The complete preparation of the UCB-PL gel required around 30 min.

### 2.3. Ulcer Management—Application of UCB-PL Gel

DFUs were first debrided to remove necrotic and infected tissues or hyperkeratotic skin. Then, normal saline was used to clean the area. Ulcer length, width and surface were measured before any study procedure. For the treatment group, the UCB-PL gel was applied to the ulcer, and then sterile gauze and non-compressible bandage were used to cover the area. This was repeated every three days for one month; the wound was irrigated with normal saline, assessed for infection, and the UCB-PL gel was applied. The control group received the SOC; including removal of necrotic, hyperkeratotic and infected tissues, cleansing the wound with normal saline and covering of the ulcer with sterile gauze and non-compressible bandage. The patients were advised to clean the ulcer with normal saline and to change the dressings daily. 

### 2.4. Follow-Up and Evaluation of the Outcome

After the first month of treatment, the patients were followed up for 20 weeks post treatment. In each visit, the study procedures included cleansing and assessment of the wound, and an interim medical history, including information regarding adverse events, concomitant medications, any possible interventions conducted, and any other issues raised in the meantime. The ulcers were photographed at two weeks, four weeks, six weeks, two months, four months and six months. 

### 2.5. Ulcer Size Assessment—imitoMeasure Application

Ulcer length, width and surface area were assessed with the imitoMeasure application (imito; imito AG, Zurich, Switzerland), using an android-based mobile device. Firstly, for the ulcer assessment, the investigators selected the legsegment in the imitoΜeasureapplication and they positioned the smartphone camera 25 cm away from and parallel to the ulcer. Afterwards, an adhesive calibration marker was placed next to the wound and a photograph was taken after recognition of the QR code by the imitoΜeasure application. If the position and the angle of the picture was not appropriate, this was recognized by the application automatically and no capture could be taken until the operator corrected the position of the QR and the camera. The calibration marker is freely accessible and must be printed by the user. The operator manually selected the borders of the wound through the photograph. The ulcer size was then calculated automatically by the application. The ulcer size was measured in each patient three times by a health care professional who was blind to the allocation arm of the participants and the mean value was used for statistical analysis.

### 2.6. Endpoints

Our primary endpoint is the percent reduction in ulcer size at the end of this study. The secondary endpoints include the assessment of the mean decrease in ulcer size during the six month-study period and the status of the wound healing at 20 weeks of follow-up, classified as either completely healed (total closure of the wound) or unhealed, and the safety of the treatment.

### 2.7. Statistical Analysis

In order to calculate the necessary number of patients, the percentage comparison formula is used, where the following values are obtained: α = predefined level of statistical significance (the most common predefined level value is α = 0.05), P = observed level of statistical significance, p1 = 0.46 (rate of effectiveness A therapeutic method), p2 = 0.79 (effectiveness rate of therapeutic method B), p2/p1 = 1.72 relative efficiency, D = p2 − p1 = 0.79 − 0.46 = 0.33, p = p1 + p2/2 = 0.625, Z (0.01 significance) = 2.576 (from related tables), and Z (power 0.80) = 0.842 (from related tables)

With a predetermined statistical significance level of α = 0.05 and a predetermined power level of 0.95, the sample size is calculated at 106 patients. In the current study, 110 patients were recruited to ensure adequacy in case any patient(s) withdrew from the study for any reason. Statistical significance tests are two-sided. In one-tailed tests, the sample size calculation estimates take values that are slightly smaller. For our research, we need to generate a set of random numbers using the statistical package SPSS and specifically the function called Rv.Uniform. This function returns a random value from a uniform distribution with a specified minimum and maximum value to generate a random number between the two limits. For our case in SPSS, we chose a minimum value of 0 and a maximum value of 1. The Rv.Uniform function produced for the 110 patients a random selection between 0 and 1. We then used the RND function in SPSS to return the rounded value between 0 and 1. Finally, we specified the treatment method with 0 or 1, respectively (1 = group to be treated − group A, 0 = control group − group B).

Data were summarized using medians with the corresponding minimum and maximum values (for continuous variables) and absolute frequencies with percentages for categorical variables. The chi-square test (or Fisher’s exact where more appropriate) was used for the comparisons of categorical variables between different groups, whereas the non-parametric Wilcoxon rank-sum test was used to assess differences in continuous variables of interest between controls and patients in the UCB-PL group. The non-parametric Friedman test was applied to identify differences in ulcer size at baseline, two weeks, four weeks, six weeks, two months, four months and six months for the effects of treatment and post hoc comparisons were performed using the Wilcoxon signed-rank test to detect differences in ulcer area between the timepoints of interest.

All tests were two sided and significance was set at the 5% level of significance. Analysis was performed using the Statistical Package for Social Sciences (SPSS) software version 23, SAS version 9.4 and the R studio 2023.09.1.

## 3. Results

### 3.1. Baseline Characteristics

In total, 120 patients were enrolled in this study. Of these, 110 patients met the inclusion/exclusion criteria of the study and were randomly assigned to the UCB-PL group (n = 52) or to the control group (n = 58). Overall, 96 patients completed the study and were included in the final analysis (Figure 1); 47 in the UCB-PL group and 49 in the control group.

Demographic data, medical status, ulcer type and ulcer characteristics at baseline are shown in Table 1. Τhe median age of participants was 63 years (range 35–85) and there was no significant difference between the groups regarding age (Wilcoxon rank-sum *p* = 0.32) and sex (chi-square *p* = 0.43). There were no statistically significant differences regarding HbA1c (Wilcoxon rank-sum *p* = 0.64), the type of diabetes (chi-square *p* = 0.21) and the duration of diabetes (Wilcoxon rank-sum *p* > 0.999). There was no difference between the groups in terms of baseline Ankle Brachial Index (ABI) (*p* = 0.66), Neurological Symptom Score (NSS) (*p* = 0.93), or Neuropathy Disability Score (NDS) (*p* = 0.15) (Table 1). 

The type of ulcer was comparable between the two groups (Fisher’s *p* = 0.66) (Table 1); however, the size of the ulcer differed significantly between the two groups, with patients in the UCB-PL group presenting a significantly wider wound area compared to those in the control group (Wilcoxon rank-sum *p* = 0.014). Approximately 23% of all participants (39.3% of informative cases) were active smokers but no significant differences regarding smoking status were observed between the two groups (*p* = 0.33). The cause of amputation was ischemia-related gangrene and the operation was combined with revascularization of the limb.

### 3.2. Outcome of Treatment

**Figure 2:**
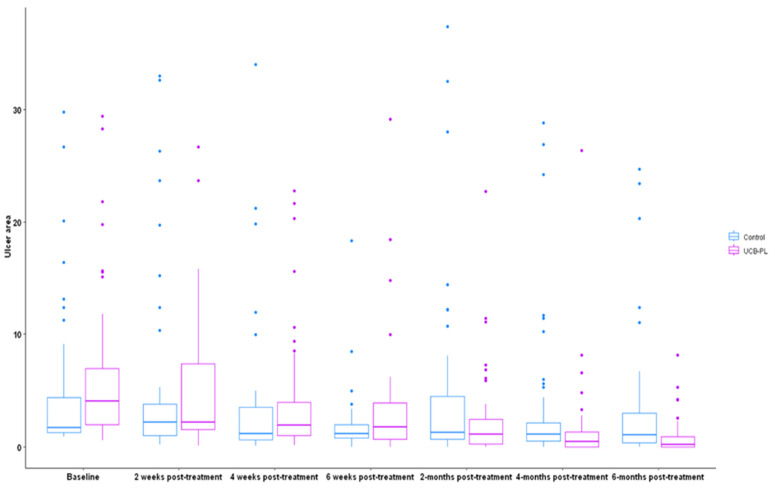
Boxplots of the distribution of ulcer area (cm^2^) per group over time.

**Figure 3:**
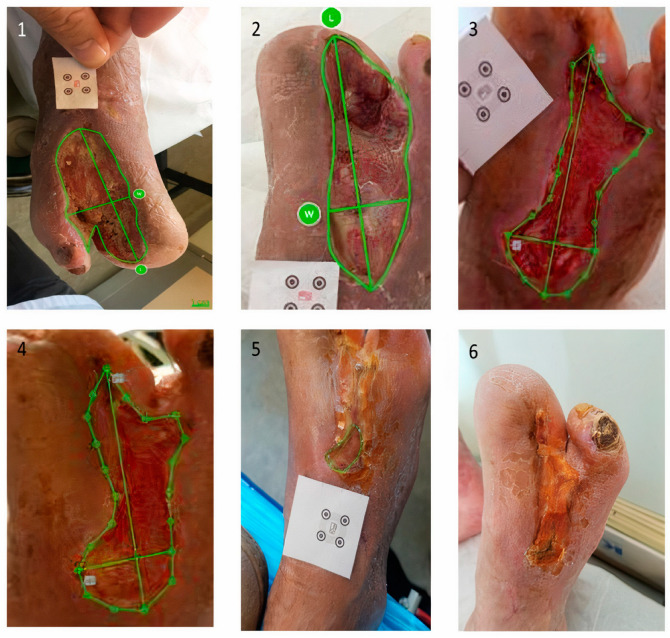
Patient in UCB-PL group: ulcer size at (**1**) baseline and the reduction in ulcer size at (**2**) two weeks, at (**3**) four weeks, at (**4**) six weeks, at (**5**) two months, and at (**6**) four months post treatment (complete healing).

The median ulcer area at baseline was 4.1 (range: 0.59–29.4) cm^2^ in the UCB-PL group and 1.7 (range: 0.90–29.8) cm^2^ in the control group. The *p*-value obtained from Friedman’s test (*p* = 0.049) indicated a marginally significant difference in the ulcer area of the two treatment groups at the different timepoints. At two and four weeks, the UCB-PL treatment resulted in a significant reduction in ulcer area (median 2.18, range: 0.13–26.7 cm^2^, Wilcoxon signed-rank *p* < 0.001 and 1.91 range: 0.14–22.76, *p* < 0.001, respectively). No change was observed in the control group compared to baseline at two weeks [2.2 (0.2–33), *p* = 0.37], whereas a significant decrease was observed at four weeks [median: 1.2 (0.11–34), *p* = 0.001]. At six weeks post treatment, patients in both groups presented a significant reduction in ulcer size compared to baseline UCB-PL 1.77 (0.9–29.1), *p* < 0.001, control group 1.2 (0–18.3), *p* < 0.001). At two, four and six months post treatment, patients on the UCB-PL treatment displayed a significant reduction in ulcer size compared to baseline (1.11 (0–22.73), *p* < 0.001, 0.5 (0–26.36), *p* < 0.001 and 0.12 (0–8.16), *p* < 0.001, respectively). Similarly, a significant reduction in ulcer size was observed in controls at two, four and six months post treatment [1.31 (0–37.4), *p* < 0.001, 1.15 (0–28.8), *p* < 0.001 and 1.05 (0–24.7) *p* = 0.002, respectively] even though the absolute difference was quite smaller compared to that observed in the UCB-PL group. (Figure 2). In total, paired data regarding ulcer area at baseline and at the end of the study were available for 78 patients (81.3%). Ulcer area was decreased at the end of the study in 67 patients (85.9%): 40 (97.6%) in the UCB-PL group, and 27 (73%) in the control group (Fisher’s *p* = 0.002). It is of note that a reduction in ulcer area at the end of the study was not achieved in only one of the informative cases in the UCB-PL group (Figure 3).

### 3.3. Complete Healing

Thirty-four (34) patients (35.4%) achieved complete healing at the end of this study, with the percentage of patients achieving complete healing being significantly higher in the UCB-PL group compared to that in the control group (55.3% vs. 16.3%, chi-square *p* < 0.001). The median time for complete healing was 16 weeks (range: 6–24) for the UCB-PL group and 24 weeks (range: 8–24) for the controls (Wilcoxon rank-sum *p* = 0.34). 

### 3.4. Safety-Adverse Events

**Figure 4:**
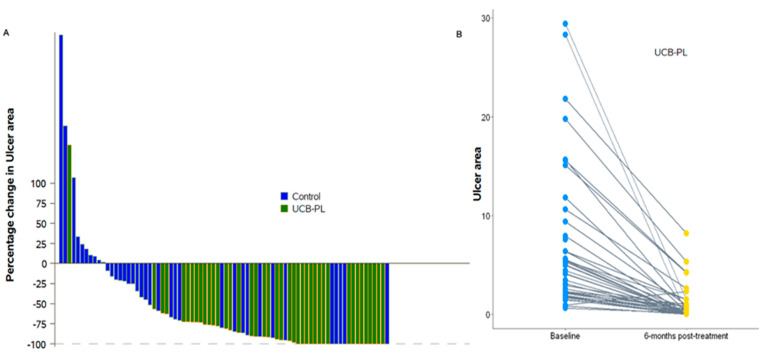
(**A**) Waterfall plot of the percentage change in ulcer area at the end of the study compared to baseline values by treatment group and (**B**) Changes in ulcer area per patient for patients in the UCB-PL group between baseline and 6 months post treatment.

**Figure 5:**
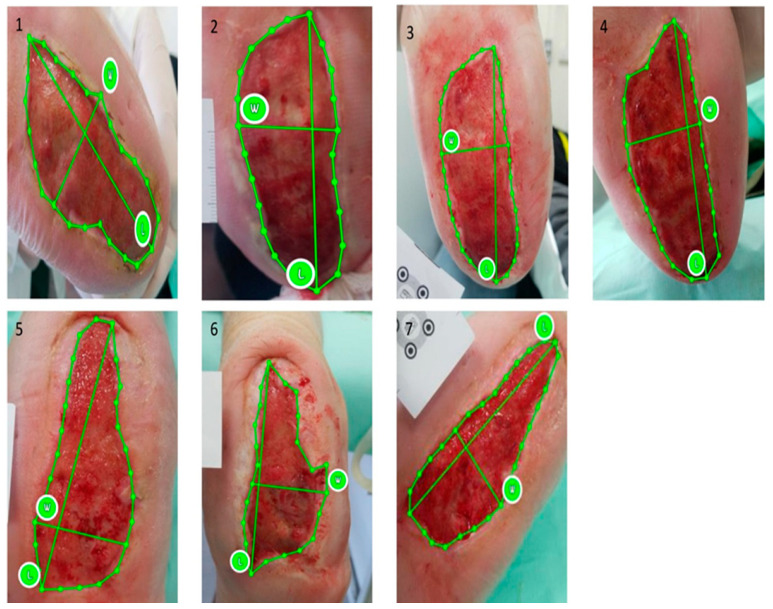
Patient in control group: ulcer size at (**1**) baseline, at (**2**) two weeks, at (**3**) four weeks, at (**4**) six weeks, at (**5**) two months, at (**6**) four months, and at (**7**) six months post treatment.

**Figure 6:**
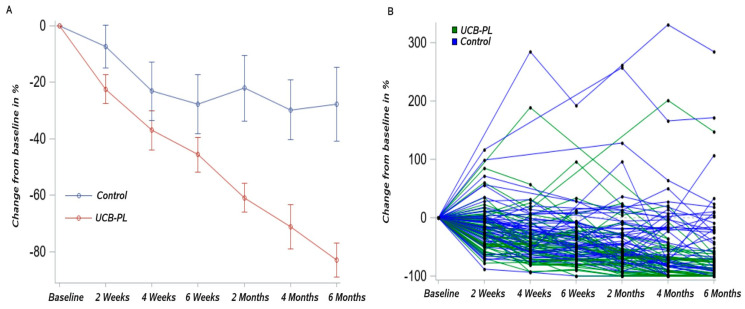
(**A**) Percentage change from baseline in ulcer area (mean ± standard error) by group. (**B**) Spider plot of percentage change from baseline in ulcer area by patient and group.

Two patients in the UCB-PL group and two patients in the control group died before the completion of this study. The deaths were neither related to the presence of diabetic foot ulcers, nor to the treatment administered. Seven patients in the control group and three patients in the UCB-PL group withdrew before the completion of this study. Adverse events were identified in twenty-one patients [UCB-PL group n = 8 (17.0%); control group n = 13 (26.5%)] at 20 weeks’ follow-up. There were no significant differences in the rate of adverse events between the two groups (chi-square *p* = 0.26). Eight patients (17%) in the UCB-PL group developed wound infections compared to 13 (26.5%) patients in the control group (*p* = 0.26). Three patients (3.1%) presented osteomyelitis, one (2.1%) patient in the UCB-PL group and two (4.1%) controls (*p* > 0.999). Moreover, two patients (2.1%) underwent minor amputation [UCB-PL group n = 1 (2.0%); control group n = 1 (2.1%), *p* > 0.999] (Figure 4, Figure 5 and Figure 6). The local application of UCB-PL was well tolerated, and no adverse events were observed during this study.

## 4. Discussion

Our study supports the hypothesis that treatment with the UCB-PL gel accelerates the healing process in comparison with SOC in patients with non-healing DFU. According to our results, the treatment with UCB-PL gel (a) led to a remarkable reduction in ulcer size, albeit not statistically significant; (b) decreased the time required for complete healing; and (c) resulted in a higher percentage of patients achieving complete healing in comparison with SOC. Furthermore, there was no significant difference between the two groups regarding adverse events, suggesting that treatment with UCB-PL is a safe therapeutic option for DFU. 

Platelets contain a substantial number of growth factors and cytokines and contribute significantly to inflammation and tissue repair. These characteristics led to the idea of using platelet-rich plasma as a therapeutic option for wound healing, particularly in patients with chronic and non-healing wounds [20,21]. Indeed, in 1986, a clinical study by Knighton et al. was the first to demonstrate that treatment with autologous platelet-derived wound-healing factors accelerate the healing process of chronic ulcers by promoting the formation and epithelization of granulation tissue [22]. After this study, a substantial number of clinical trials showed the favorable effect of platelet-rich plasma on wound/ulcer healing, whether diabetic or not in origin [23,24,25]. More recently, several studies have evaluated the efficacy of PL, a platelet derivative rich in cytokines and growth factors, in the wound-healing process [26,27]. Barsotti et al. [26] evaluated the in vitro effect of PL on the proliferation and activity of various cell types (endothelial cells, monocytes, fibroblasts and keratinocytes). According to the results, PL induced viability, proliferation, cell migration and angiogenic activity (only in the highest concentration). Losi et al. [27] showed that the application of bilayered fibrin/poly(ether)urethane scaffold loaded with PL in full-thickness skin wounds of diabetic mice significantly accelerated wound closure. Jafar et al. [28] reported two cases in which treatment with injections of human PL promoted human keratinocyte migration and resulted in the complete healing of previously un-healed DFU at 8 weeks post-treatment. In a meta-analysis of 20 trials, the use of autologous PRP increased complete wound closure, shortened the healing time, and reduced the wound size in individuals with diabetic ulcers in contrast to venous or pressure ulcers, where the evidence was insufficient [29]. However, in a study by Moneib et al. [30], where 40 patients with chronic venous leg ulcers were included, the mean percentage improvement in the area of the ulcer post PRP and conventional therapy was 67.6%, 36.6% and 13.67%, respectively, and such beneficial results were reproduced in another study by Helmy et al. [31] with a comparable study sample. It is worth mentioning that the beneficial results of PRP are also related to the form of administration; in a study by Elbarbary et al. [32], PRP injection promoted healing more than PRP application and compression.

The PRP and other platelet derivatives (e.g., PL) used in the majority of current clinical trials are obtained from autologous peripheral blood; this inevitably leads to practical and clinical limitations, as the amount of autologous PRP is limited and affected by inter-individual variability [33,34]. The use of allogenic blood products has been demonstrated to overcome these limitations. Moreover, UCB-PL gel has a higher concentration of growth factors compared to gel obtained from adult platelets. Parazzi et al. [35] have shown that cord blood platelet gel releases a substantial number of growth factors such as platelet-derived growth factor-BB transforming growth factor-b1 and fibroblast growth factor-b. In addition, Losi et al. [36] showed that human UCB-PL contains high levels of pro-angiogenic growth factors which may improve the viability, proliferation, and cell migration, suggesting UCB-PL as an effective tool for wound healing. According to clinical studies, the use of UCB-PL for wound healing has encouraging outcomes. A pilot study from Tadini et al. demonstrated the beneficial effect of UCB-PL in the treatment of dystrophic epidermolysis bullosa skin lesions in three children [37]. However, the evidence regarding the efficacy of UCB-PL in DFU is scarce. A pilot clinical trial showed the efficacy and safety of cord blood platelet gel in three patients with DFU [38]. Volpe et al. [39] conducted the first randomized clinical trial evaluating the effect of platelet gel derived from UCB in twenty patients with diabetes after lower limb revascularization. According to the results, treatment with UCB platelet gel led to a mean ulcer area reduction of 76% compared to 49% in the control group (*p* < 0.01). 

The major strengths of our study are the large size of the sample and its prospective randomized design. The use of the imitoMeasure application to assess ulcer size adds to the objectivity and credibility of the assessments. The traditional measurements of ulcer size (e.g., paper ruler) are an accurate tool for determining length and width; however, the obtained data regarding the ulcer area are less reliable due to morphologic irregularities. In addition, digital measuring systems are versatile tools that can be easily applied in daily clinical practice. According to research data, the imitoMeasure application is a useful and practical smartphone app with excellent accuracy and reproducibility [40,41]. However, a limitation is that this was an open-label study because, due to the nature of UCB-PL, it was not possible to have a similar placebo formulation. However, the estimation of ulcer dimensions was carried out three times by a person who was blinded to the allocation group, which limits the possibility of systemic errors. The larger ulcer area at the baseline inclusion is not an advantage for the cord blood platelet lysate group but, according to our statistical analysis, it has been considered that the difference in ulcer size does not affect the results.

Our results demonstrated that the use of UCB-PL gel as an add-on treatment to the clinical standard of care results in a significant mean reduction in ulcer area and accelerates the healing process.

## 5. Conclusions

This randomized controlled clinical trial demonstrates the efficacy and safety of UCB-PL for the treatment of diabetic foot ulcer. The data suggest the use of UCB-PL as an adjunctive therapeutic tool in the management of diabetic foot ulcer. More randomized controlled clinical trials are needed to confirm our results.

## Figures and Tables

**Table 1:** Baseline characteristics: UCB-PL group vs. control group.

	Total (n = 96)	UCB-PL Group (n = 47)	Control Group (n = 49)	*p*-Value
**Age**, years (n = 75)	63 (35–85)	70 (48–75)	61 (35–85)	0.32
**Sex**				0.43
Male Female,	70 (72.9) 26 (27.1)	36 (76.6) 11 (23.4)	34 (69.4) 15 (30.6)	
**Type of diabetes**				0.21
Type 1 DM Type 2 DM	17 (17.7) 79 (82.3)	6 (12.8) 41 (87.2)	11 (22.4) 38 (77.6)	
**Diabetic Nephropathy**				0.29
Yes	24 (25.0)	14 (29.8)	10 (20.4)	
No	72 (75.0)	33 (70.2)	39 (79.6)	
**Diabetic Retinopathy**				0.29
Yes	24 (25.0)	14 (29.8)	10 (20.4)	
No	72 (75.0)	33 (70.2)	39 (79.6)	
**Diabetic Neuropathy**				0.84
Yes	46 (47.9)	23 (48.9)	23 (46.9)	
No	50 (52.1)	24 (51.1)	26 (53.1)	
**Intervention prior to randomization**				0.72
Yes	23 (24.0)	12 (25.5)	11 (22.4)	
No	73 (76.0)	35 (74.5)	38 (77.6)	
**Smoke** (n = 56)				0.33
Yes	22 (39.3)	10 (33.3)	12 (46.2)	
No	34 (60.7)	20 (66.7)	14 (53.8)	
**HbA1C (%)** (n = 45)	8.2 (5.4–13.8)	8.2 (5.7–13.4)	7.8 (5.4–13.8)	0.64
**DM duration, years** (n = 49)	27.0 (1.00–60.0)	20.0 (2.0–40.0)	27.0 (1.00–60.0)	>0.999
**ABI** (n = 59)	1.06 (0.33–2.2)	1.03 (0.33–2.2)	1.06 (0.37–1.9)	0.66
**NSS** (n = 65)	0.00 (0.00–9.0)	0.00 (0.00–9.0)	1.5 (0.00–9.0)	0.93
**NDS** (n = 63)	9.0 (0.00–10.0)	9.0 (0.00–10.0)	8.0 (2.0–10.0)	0.15
**Type of ulcer**	0.66			
Neuropathic ulcer	51 (53.1)	22 (46.8)	29 (59.2)	
Ischemic Ulcer	25 (26.0)	13 (27.7)	12 (24.5)	
Neuropathic/Ischemic Ulcer	12 (12.5)	7 (14.9)	5 (10.2)	
Ulcer after amputation n	8 (8.3)	5 (10.6)	3 (6.1)	

Data are presented as median (min–max) or number (percentage), DM: diabetes mellitus; HbA1c: hemoglobin A1c; ABI: Ankle Brachial Index; NSS: Neurological Symptom Score, NDS: Neuropathy Disability Score.

## Data Availability

The data presented in this study are available on request from the corresponding author. The data are not publicly available due to local privacy and data protection regulations.
